# Beyond Attributable Burden: Estimating the Avoidable Burden of Disease Associated with Household Air Pollution

**DOI:** 10.1371/journal.pone.0149669

**Published:** 2016-03-16

**Authors:** Randall Kuhn, Dale S. Rothman, Sara Turner, José Solórzano, Barry Hughes

**Affiliations:** 1 Global Health Affairs Program, Josef Korbel School of International Studies, University of Denver, Denver, Colorado, United States of America; 2 Frederick S. Pardee Center for International Futures, Josef Korbel School of International Studies, University of Denver, Denver, Colorado, United States of America; Indian Institute of Toxicology Research, INDIA

## Abstract

**Background:**

The Global Burden of Disease (GBD) studies have transformed global understanding of health risks by producing comprehensive estimates of attributable disease burden, or the current disease that would be eliminated if a risk factor did not exist. Yet many have noted the greater policy significance of avoidable burden, or the future disease that could actually be eliminated if a risk factor were eliminated today. Avoidable risk may be considerably lower than attributable risk if baseline levels of exposure or disease are declining, or if a risk factor carries lagged effects on disease. As global efforts to deliver clean cookstoves accelerate, a temporal estimation of avoidable risk due to household air pollution (HAP) becomes increasingly important, particularly in light of the rapid uptake of modern stoves and ongoing epidemiologic transitions in regions like South and Southeast Asia.

**Methods and Findings:**

We estimate the avoidable burden associated with HAP using International Futures (IFs), an integrated forecasting system that has been used to model future global disease burdens and risk factors. Building on GBD and other estimates, we integrated a detailed HAP exposure estimation and exposure-response model into IFs. We then conducted a counterfactual experiment in which HAP exposure is reduced to theoretical minimum levels in 2015. We evaluated avoidable mortality and DALY reductions for the years 2015 to 2024 relative to a Base Case scenario in which only endogenous changes occurred. We present results by cause and region, looking at impacts on acute lower respiratory infection (ALRI) and four noncommunicable diseases (NCDs). We found that just 2.6% of global DALYs would be averted between 2015 and 2024, compared to 4.5% of global DALYs attributed to HAP in the 2010 GBD study, due in large part to the endogenous tendency towards declining traditional stove usage in the IFs base case forecast. The extent of diminished impact was comparable for ALRI and affected NCDs, though for different reasons. ALRI impacts diminish due to the declining burden of ALRI in the base case forecast, particularly apparent in South Asia and Southeast Asia. Although NCD burdens are rising in regions affected by HAP, the avoidable risk of NCD nonetheless diminishes due to lagged effects. Because the stove transition and the decline of ALRI are proceeding more slowly in Sub-Saharan Africa, avoidable impacts would also be more persistent (3.9% of total DALY due to HAP) compared to South Asia (3.6%) or Southeast Asia (2.5%).

**Conclusions:**

Our results illustrate how a temporal dynamic calculation of avoidable risk may yield different estimates, compared to a static attributable risk estimate, of the global and regional burden of disease. Our results suggest a window of rising and falling opportunity for HAP interventions that may have already closed in Southeast Asia and may be closing quickly in South Asia, but may remain open longer in Sub-Saharan Africa. A proper accounting of global health priorities should apply an avoidable risk framework that considers the role of ongoing social, economic and health transitions in constantly altering the disease and risk factor landscape.

## Introduction

The Global Burden of Disease (GBD) studies and the associated Comparative Risk Assessments (CRA) have transformed global understanding of health risks [1]. CRA played an especially important role in drawing attention to risk factors like household air pollution (HAP) that are associated with multiple sequelae, and thus not easily appreciated through casual observation [1,2]. CRA estimates of attributable burden, defined as the “current and/or future burden of disease if past exposure to a risk factor had been equal to some counterfactual distribution” [3]. Yet many have noted the greater policy significance of calculating *avoidable burden*, defined as “the reduction in the *future* burden of disease if the *current or future exposure* to a risk factor is reduced to a counterfactual distribution” [3] (emphasis added). Avoidable risk may be considerably lower than attributable risk if baseline levels of exposure or disease are declining, or if a risk factor carries lagged effects on disease. We use the International Futures (IFs) integrated forecasting system to estimate avoidable impacts of the total elimination of HAP on future mortality risks, thereby demonstrating the broader importance of estimating avoidable risk.

The past quarter-century has seen an evolving understanding of the significance of HAP for communicable and noncommunicable respiratory and cardiovascular conditions [4–14]. Based on mean risk factor estimates, GBD 2010 estimated a population attributable risk (PAR) for HAP of 111 million disability-adjusted life years (DALYs), or 4.5% of the global total, making it one of the three leading risk factors [2,15].

Yet, PAR estimations can change significantly with the passage of time. GBD 2010’s estimate of 4.5% of global DALYs attributable to HAP in 2010 represents a 37% decline from the 7.0% of DALYs attributable to HAP in 1990. Over the same period, the DALY share of risks associated primarily with communicable disease and poverty-related risk factors declined even more, as in the case of childhood underweight (-64%) and suboptimal breastfeeding (-62%), while risks principally associated with non-communicable disease (NCD) and affluence-related risk factors increased, as in high body mass index (+51%) and high fasting plasma glucose (+41%) [16].

HAP is more complicated. A growing share of households globally are adopting gas or electric stoves, an income- and development-related transition that will reduce the HAP-related disease burden even without global action[17–19]. HAP affects one disease that is rapidly declining (ALRI) but also four NCD risks that are rising in most regions, making it difficult to infer changes in baseline disease burden over time. The rise of NCD risk factors like ambient air pollution, smoking, and aging may wipe away some of the benefits of HAP reduction. Effects on reduced NCD risk also carry time lags that are not represented in attributable risk calculations [13,19,20].

We estimate the avoidable burden associated with HAP using IFs, an integrated forecasting system that has been used to model future global disease burdens and risk factors [21,22]. Building on GBD and other estimates, we integrated a detailed HAP exposure estimation and exposure-response model into IFs. We then conducted a counterfactual experiment in which HAP exposure is reduced to theoretical minimum levels in 2015. We evaluated avoidable mortality and DALY reductions for the years 2015 to 2024 relative to a Base Case scenario in which only endogenous change in disease occurred. While our work speaks to the value and regional focus of HAP efforts, it carries broader implications for any efforts, global or domestic, to quantify disease control priorities under changing baseline conditions.

## Methods

GBD 2010 included comprehensive estimates of deaths and DALYs attributable to 67 modifiable risk factors for 291 causes of disease and injury [1]. The risk factor assessment was based on the calculation of population attributable risk (PAR) and population attributable fraction (PAF). For each cause of death and disability-adjusted life year (DALY) associated with a given risk factor, PAR is calculated as the total burden of death/disease could be eliminated if exposure levels were reduced to the theoretical minimum, while PAF is the share of total disease burden that could be eliminated, calculated as

$$PAR=\sum RR\left(x\right)P\left(x\right)-\sum RR\left(x\right)P^{\prime }\left(x\right)$$ (1)

$$PAF=\;\frac{\sum RR\left(x\right)P\left(x\right)-\sum RR\left(x\right)P^{\prime }\left(x\right)}{\sum RR\left(x\right)P\left(x\right)}=\frac{PAR}{Total\;Burden}$$ (2)

*where* RR(x) is the relative risk associated with exposure level x, P(x) is the prevalence of risk factor x in each country/sex/age group, and P’(x) is the theoretical minimum level of risk factor exposure in each country/sex/age group. PARs can be summed across cause, age and sex subcategories to produce aggregate results, e.g., total global mortality attributable to a given risk factor. We situate a temporal version of this approach into the International Futures integrated forecasting system to estimate avoidable risk.

### International Futures (IFs)

**Fig 1:**
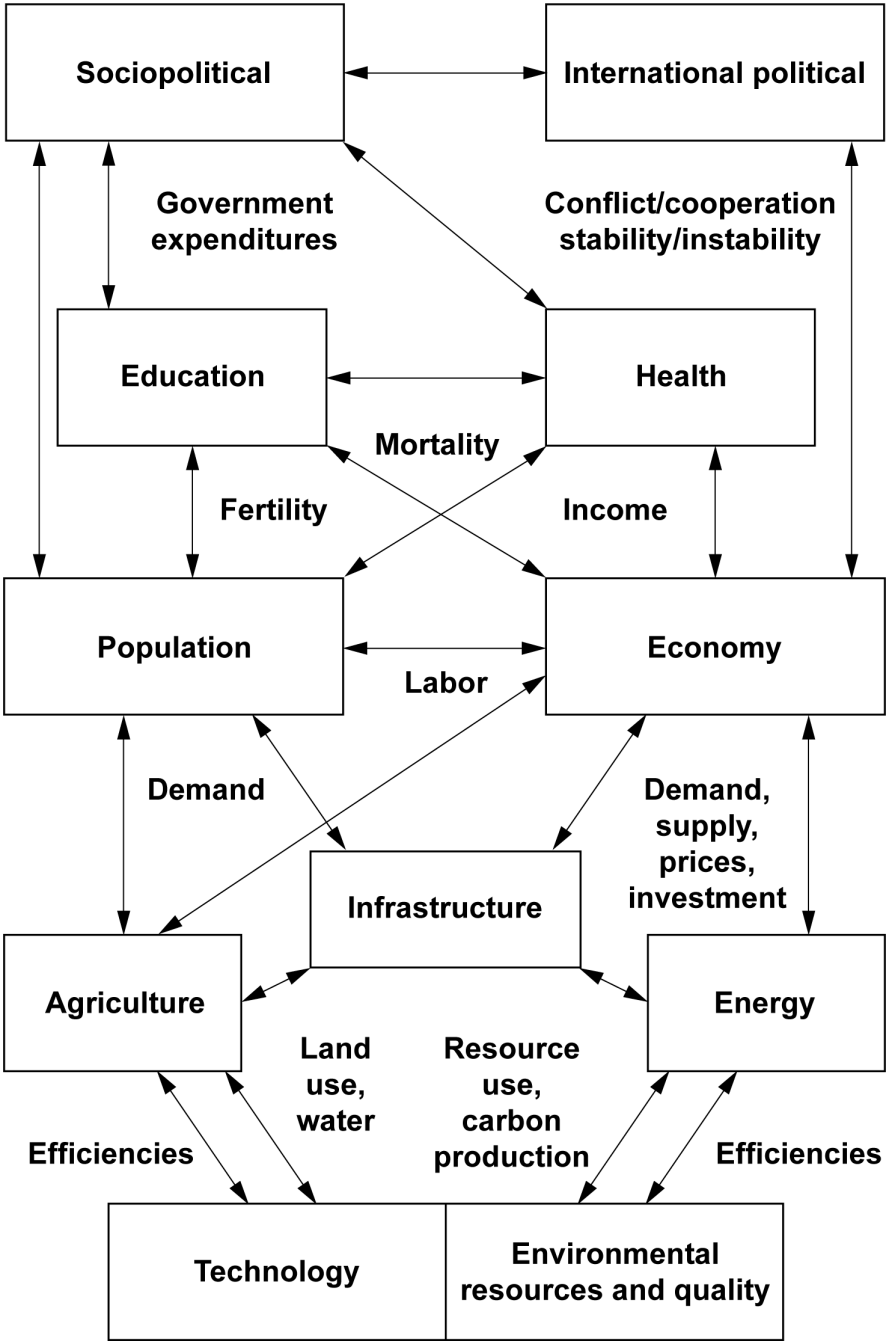
The major modules of International Futures (IFs). Note: Links shown are examples from a much larger set of linkages.

The International Futures (IFs) simulation system is a structure-based, agent-class driven, dynamic modeling tool [21,22]. Fig 1 shows the major models in the system, each drawing upon standard modeling approaches from a wide range of disciplines. All outcomes including health and key risk factors are forecast jointly as part of the integrated framework. We describe our basic approach to representing and forecasting stoves and mortality, with further detail in S1 Appendix.

#### IFs health model

The IFs health model uses distal drivers of socioeconomic change and proximate risk factors like traditional stove use to forecast changing mortality and disease burdens [23,24]. Data on 15 causes of death by country, age, and sex come from the World Health Organization’s 2010 Global Health Estimates (GHE), and are broadly similar to the estimates constructed by the Institute for Health Metrics and Evaluation for the GBD 2010. The IFs health forecast has been published extensively and assessed through internal and external validation exercises against historical data and UN Population Division all-cause mortality forecasts [21–24]. Forecasts based on distal socioeconomic drivers build on the World Health Organization’s 2004 Global Burden of Disease forecasts, predicting age- sex- cause-specific mortality as a function of GDP per capita, total years of education (for adults 25 and older), a smoking impact factor, and time. The age-sex-specific nature of the IFs population module allows us to calculate DALYs averted from deaths averted.

After initialization, subsequent changes in cause-specific mortality rates are driven by the distal driver regressions and by adjustments to account for leading risk factors. The proximate risk factor adjustment in a given forecast year results from a comparison of the expected PAFs from a model based only on distal drivers versus one based on a more extensive set of drivers known to affect the risk factor, as described for traditional and modern cookstoves below. The adjustment takes the following form:

$$Mortality_{Final}=Mortality_{Distal}*\frac{\left(1-PAF_{Distal}\right)}{\left(1-PAF_{Full}\right)}$$ (3)

The risk factor distribution and thus the PAF adjustment can also be manipulated exogenously, allowing us to introduce an experiment in which solid fuel use and HAP exposure are reduced to theoretical minimum levels in 2015.

#### IFs stove model

The IFs stove model represents national trajectories in the use of traditional and modern stoves. We drew historical data on the percentage of households in each country primarily using solid fuels from the 1990 to 2010 country-level dataset from UNSTATS and WHO used in GBD 2010 [25,26]. We used evidence of historic changes between 1990 and 2010 to develop a logistic function forecasting increased use of modern stoves to changes in income, urbanization, and electrification [24,27]:

$$z=3.40-0.128*GDPPC-0.037*Elec-0.011*Urban\;Stove=\frac{100}{e^{-z}+1}$$ (4)

Where *Stove* is the expected share of the population using traditional stoves, *GDPPC* is the per capita gross domestic product, *Elec* is the percent of households with access to electricity, and *Urban* is the percent of households living in urban areas. We use the same equation to fill in missing values for 2010.

#### Relating traditional stove use to HAP exposure

To estimate the consequences of changes in traditional stove use on HAP exposure and health risks, we conducted a review of the relationship between cookstove type and PM_2.5_ exposure, drawing heavily on literature used by GBD 2010. Our review included studies that measured concentration levels by stove type (not fuel type) in relation to PM_2.5_ (or a measure that could be converted to PM_2.5_) and provided evidence on exposure variation by locations in the home (see Bibliographic Table for Exposure Estimation for full list of papers reviewed). We then estimated PM_2.5_ exposure levels as a function of cookstove type, gender, and age, following the EPA guidelines for exposure assessments, using the following equation [28–30]

$$E_{C}=\Sigma_{i=1}^{n}E_{i}*T_{i}$$ (5)

Where E_c_ = average daily exposure concentration for an individual, E_i_ = average daily exposure concentration in microenvironment i by cookstove type, and T_i_ = fraction of 24 hour day spent in microenvironment i.

Based on these calculations, and with some qualitative assessment, we calculated separate exposure levels for women age 25+, men age 25+, and children under age 5. We calibrated our exposure parameters to those used in the Household Air Pollution Intervention Tool (HAPIT) [31]. We tested a wide variety of alternate base case exposure measures, which had minimal effect on our results. There was greater heterogeneity in the estimation of sex differences in exposure, and our results focus on the all-sex findings.

#### Exposure-response relationships

GBD 2010 developed integrated exposure response (IER) curves for the effects of HAP on acute lower respiratory infections (ALRI) occurring among children under 5 and five causes among adults age 25+: chronic obstructive pulmonary disease (COPD), ischaemic heart disease (IHD), cerebrovascular disease (CVD), cancers of the trachea, bronchus and lung (lung cancers), and cataracts [1,2,7,11,13,28,32]. We used these IER curves to convert exposure levels into disease-specific mortality risks, with the relative risks by age group and sex shown in Table A1 in S1 Appendix [32]. Because IFs represents 15 large causes of death/disability rather than the 291 specific causes used by GBD 2010, we separated HAP-related causes of death out from their larger cause groups in 2010 based on the distribution of total deaths for the WHO subregion (specifically, ALRI was subdivided from respiratory infections, IHD and CVD from cardiovascular disease, COPD from respiratory illness, and lung cancers from malignant neoplasms). In all cases except lung cancers, the HAP-affected sub-cause accounted for a substantial share of the total cause group, thereby minimizing the potential error introduced by this assumption.

Finally, we introduced a simplified lagged impact structure modified from Environmental Protection Agency (EPA) guidelines[30]. We assumed that the reduction in ALRI risks happened immediately; for CVD we assumed a two year lag, and for pulmonary diseases five years [33].

## Results

### Base Case Stove Forecast

**Fig 2:**
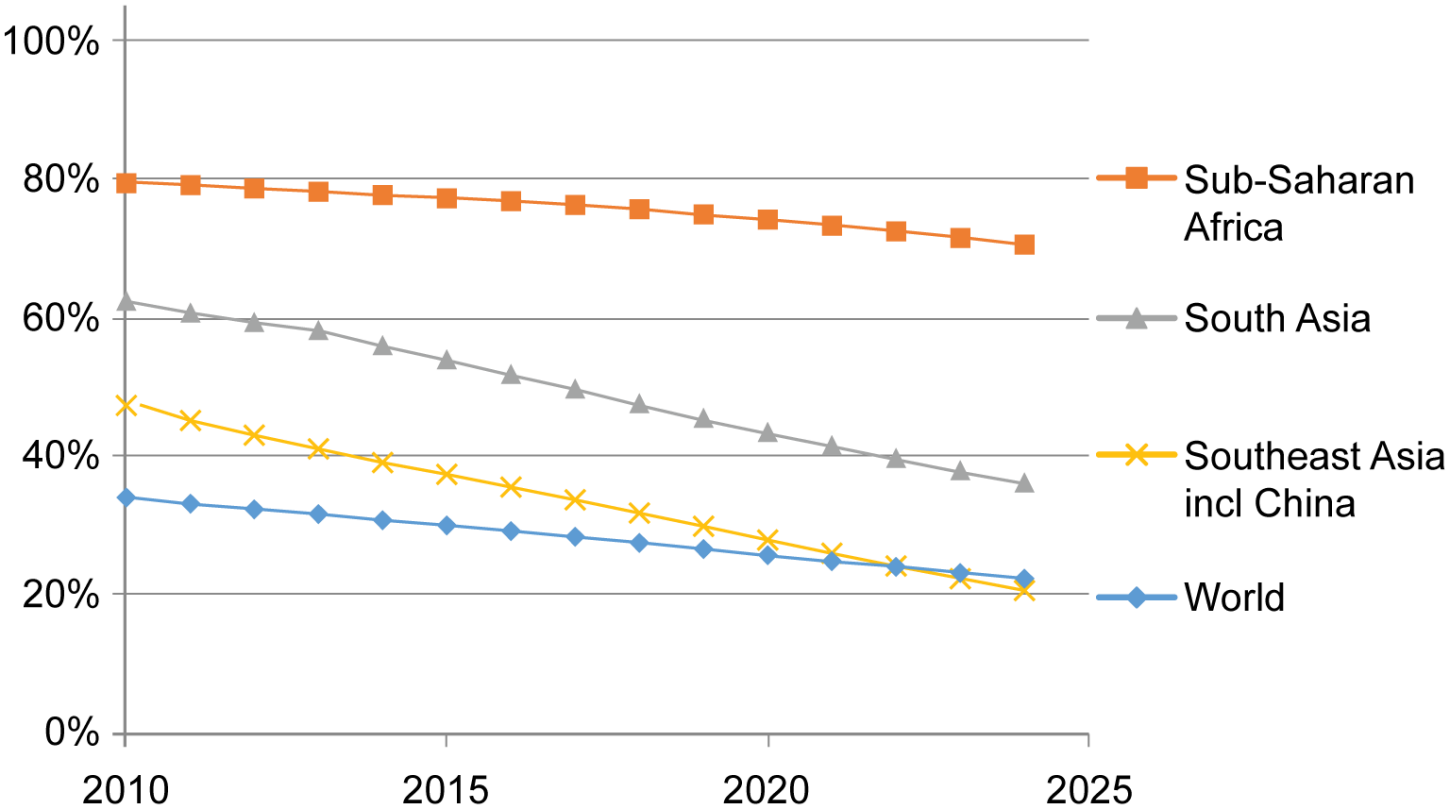
Base case forecast of prevalence of household solid fuel exposure, 2010–2024, World and key GBD super-regions. Source: IFs version 7.01.

The IFs Base Case anticipates continued rapid reductions globally in the share of households using solid fuel, from 34% to 22% between 2010 and 2024, as shown in Fig 2. In Sub-Saharan Africa (SSA), initial solid fuel use is high at 79% and is expected to decline only to 71% by 2024. In Southeast Asia including China (SEA), solid fuel use was at 47% in 2010 and is forecast to decline to 21% by 2024. While South Asia (SAS) had relatively high HAP use in 2010 at 62% of households, this is expected to decline to just 36% in 2024 due to rapid economic growth, urbanization, and electrification.

### Base case forecast for HAP-affected disease

**Fig 3:**
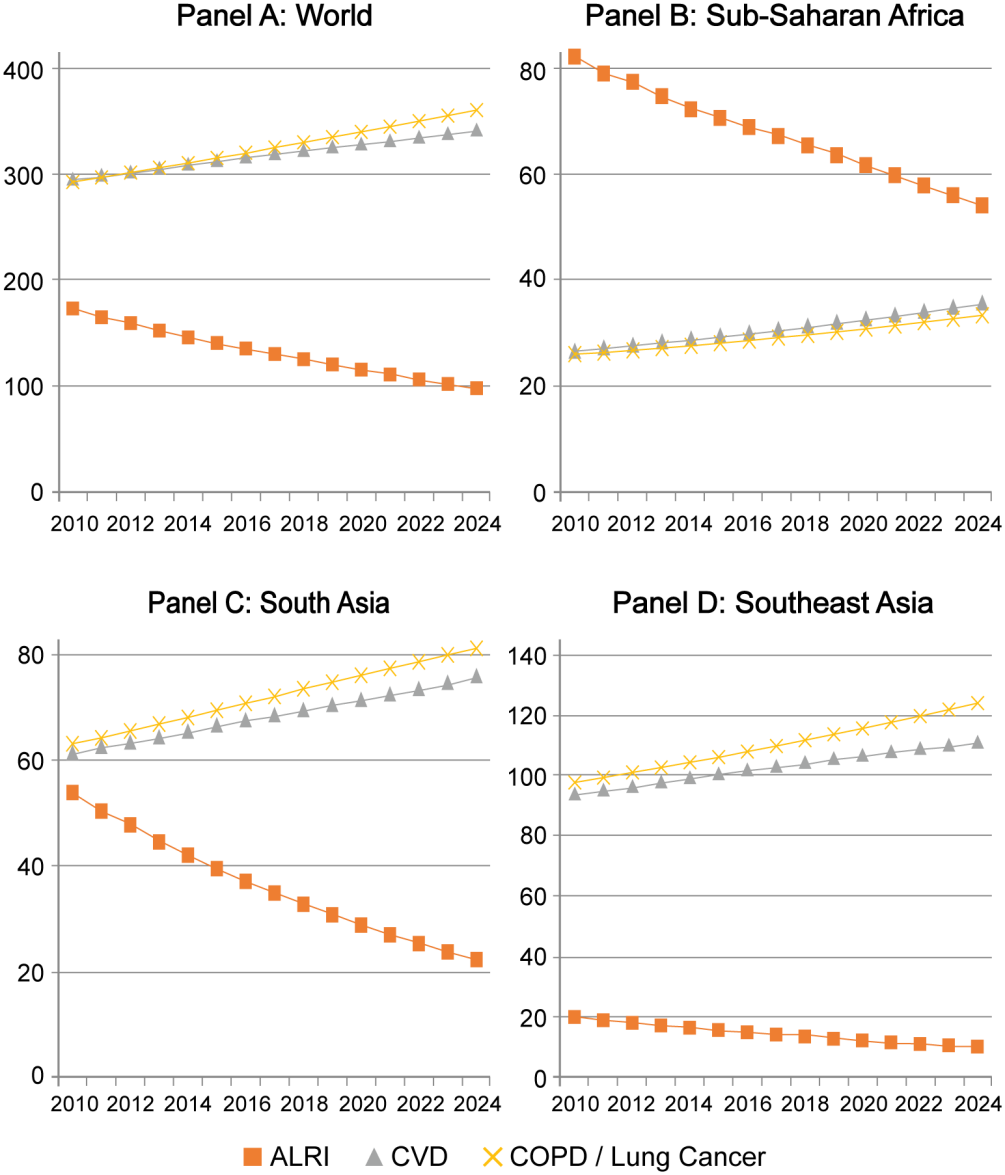
Base Case forecast of annual DALYs due to HAP-related causes, 2010–2024, World and key GBD super-regions. Source: IFs version 7.01.

At the same time, key sequalae of HAP vary in level and trend across regions. Fig 3 presents the Base Case trend in DALYs for the three main sets of diseases affected by HAP: ALRI among children age 0–4, CVD, and lung conditions including COPD and lung cancer. In 2010 ALRI accounted for 174 million DALYs, or 5.9% of all DALYs worldwide, but the Base Case expects this impact to decline to 98 million DALYs, or 3.6% of the total, by 2024. CVD and lung cancer already carried a more substantial burden of DALYs, with the burden set to gradually rise.

In SSA, which has seen the least progress in its epidemiologic transition, there remains a high burden of ALRI, which currently accounts for a greater burden of DALYs than CVD, COPD, and lung cancer combined. Though the Base Case forecast anticipates considerable decline in ALRI burden in SSA, the rate of decline will be substantially slower than in other regions. Even in 2024, 44% of all HAP-affected DALYs (54 out of 122 million) would result from ALRI. SAS is midway through its epidemiologic transition. CVD and respiratory conditions already each account for more DALYs than ALRI, and the Base Case shows that gap widening considerably over the next decade. By 2024, the model anticipates that only 12% of all HAP-affected DALYs (22 out of 180 million) in SAS will result from ALRI. Finally, SEA is well along in its transition, with high and rising CVD, lung cancer, and COPD burdens. ALRI accounted for just 9% of HAP-related DALYs in 2010, and the Base Case sees this declining to 4% by 2024.

### HAP elimination experiment results

**Fig 4:**
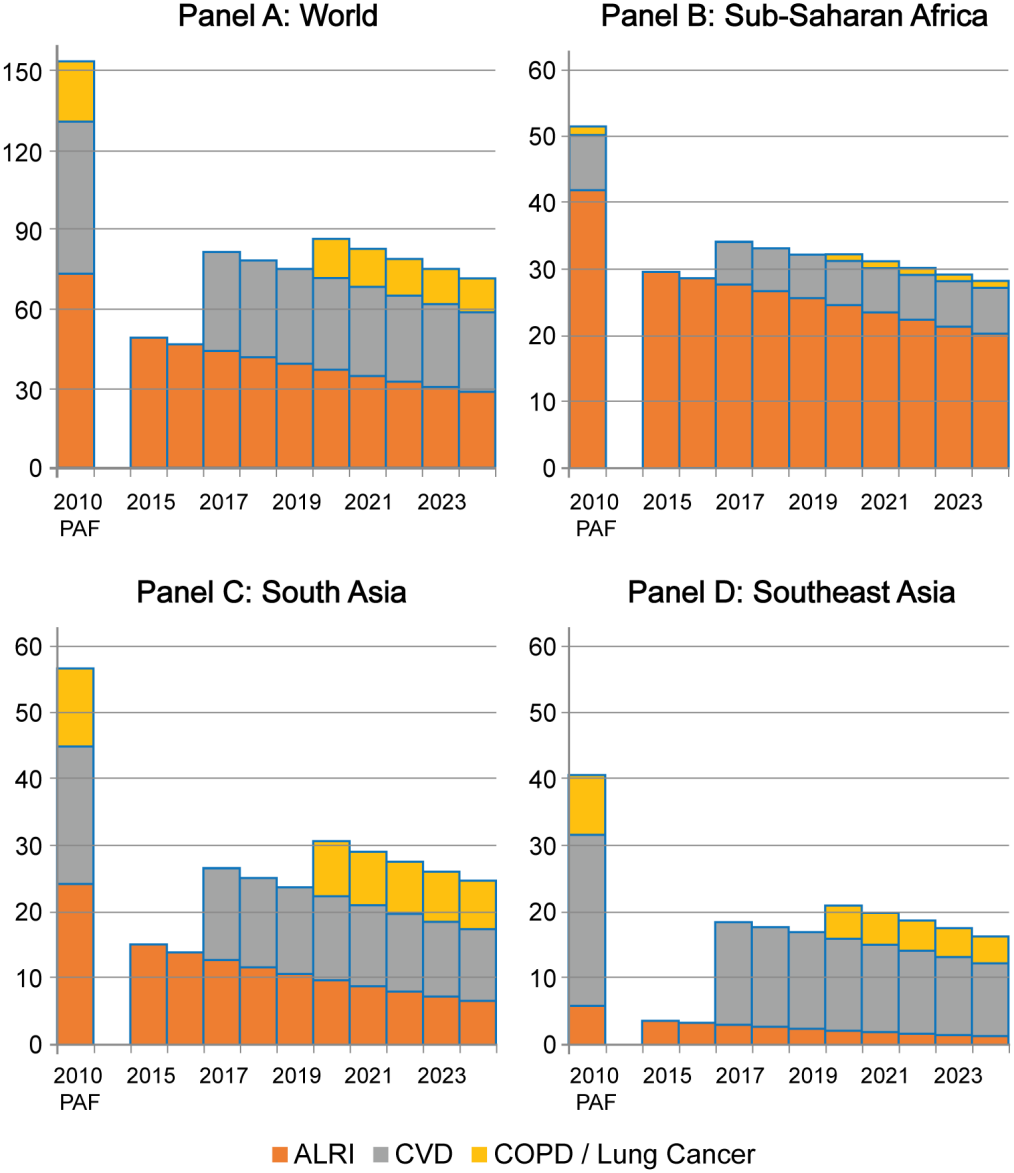
Annual DALYs averted due to theoretical minimum reduction in Household Air Pollution Exposure, 2015–24 forecast compared to 2010 Attributable Risk Calculation. Source: IFs version 7.01

Fig 4 reports results of the theoretical minimum cause-elimination experiment conducted in IFs. Impacts on ALRI begin immediately in 2015, followed by impacts on CVD in 2017 and COPD/lung cancer impacts in 2019. In order to observe differences between attributable and avoidable risk estimates, we compare the results of our experiments to attributable risk estimates based a GBD-type calculation using IFs disease and stove data for 2010. Globally, the annual DALYs averted via the global elimination of HAP would amount to 73 million, or less than half of the 154 million DALYs based on an attributable risk calculation. This difference is due in part to the rapidly diminishing burden of ALRI. While we estimate that elimination of HAP-related ALRI in 2010 could have averted 73 million DALYs, the possible DALYs averted will have dropped to 49 million in 2015 and will decline further to 29 million in 2024.

Avoidable DALYs due to non-communicable disease are reduced to a similar degree. Although the baseline burdens of CVD and lung conditions are rising, the most rapid growth is likely in SEA and SAS, which are also seeing the rapid adoption of liquid fuels and the rise of other NCD risk factors that swamp the effects of HAP reductions. As a result, while we attribute 58 million CVD DALYs to HAP in 2010, only 30 million would actually be averted by 2024. COPD DALYs go from an attributable burden of 23 million in 2010 to just 13 million DALYs averted in 2024.

The results for specific regions further illustrate the gap between avoidable and attributable risk. In SSA, because the rate of endogenous stove adoption is lower and the rate of ALRI decline slower, a greater share of the potential ALRI impact endures over time. In 2024, 48% of the 2010 attributable risk of ALRI remains in place for SSA (20 million in 2024 vs. 42 million in 2010), while only 27% remains for SAS (6.6 million vs. 24.1 million) and 22% for SEA (1.3 million vs. 3.4 million). Because ALRI accounts for such a disproportionately large burden of disease in SSA, the enduring ALRI effects alone make the avoidable risk associated with HAP substantially greater in SSA than in the other regions.

NCD burdens also favor enduring impact in SSA. While potential impacts of HAP elimination on CVD and lung conditions persistently decline in SAS and SEA, the forecast actually suggests that the number of CVD DALYs averted will be rising in SSA, due both to rising NCD burdens and relatively young populations that are not yet exposed to competing NCD risks.

## Discussion

A temporally dynamic calculation of avoidable risk provides a markedly different view of disease control priorities than a static estimate of attributable risk. GBD 2010 attributed 4.5% of global DALYs to HAP. Our comparable estimate using 2010 IFs data suggested that 5.3% of DALYs are attributable to HAP, yet we found that only 2.6% of DALYs could actually be avoided from 2015 to 2024 after accounting for changing disease burdens, endogenous adoption of liquid fuels, lagged effects of HAP reduction, and competing mortality risks.

Whereas CRA suggests that South and Southeast Asia face the greatest burden of deaths and DALYs attributable to HAP, we find that even over a relatively brief 10-year horizon the avoidable burden will shift dramatically to Sub-Saharan Africa. We estimate that the total elimination of HAP in 2015 would, between 2015 and 2024, result in 31.6 million DALYs averted in Sub-Saharan Africa (3.9% of the total DALY burden) compared to 24.6 million in South Asia (3.6% of the total) and 14.8 million in Southeast Asia (2.5% of the total). This difference stems for the most part from the faster pace of ongoing liquid stove adoption in South and Southeast Asia. It also stems from the more rapid transition in these regions away from ALRI risks, which have strong associations to HAP, to NCD risks that are subject to longer lags and to the rise of other NCD risk factors.

While we hope that this study has made a significant contribution to comparative risk assessment, it has limitations. First, in building on CRA, our study carries many of its same weaknesses, including uncertainty in our risk exposure estimates; the lack of variation in RRs by region and over time; and the lack of attention to interactions between HAP risks and related risk factors like smoking and outdoor air pollution. Second, IFs mortality projections forecast 15 major cause groups, such as respiratory infections, chronic respiratory diseases, and CVD, rather than the specific sub-causes used in the 2010 GBD. Finally, our model includes a simplified representation of household combustion and air pollution. While our modern stove adoption trajectory is based on historical evidence, it remains the case that our results are sensitive to variations in the pace of modern stove uptake.

In spite of these limitations, what emerges from our analysis is a story of an unfolding epidemic, with key lessons for the campaign to reduce HAP exposure and for broader efforts to quantify disease control priorities. The window for low-cost public action against HAP may have already closed in Southeast Asia, may be closing quickly in South Asia, and is open for a relatively short period in Sub-Saharan Africa. Policymakers are currently making decisions about the rollout of moderately efficient “clean” solid fuel stoves that can be distributed to individual households versus the rollout of gas and electric fuel grids with high fixed costs. Standalone solutions may prove attractive in Sub-Saharan Africa since they can be rolled out quickly with limited public sector involvement, and even modest HAP reductions can reduce the persistent burden of ALRI. Other regions may need more costly liquid-fuel solutions accompanied by regulatory efforts to control parallel risks associated with ambient air pollution and tobacco smoke.

Within a dynamic temporal framework we may approach HAP or any other health problem in terms of windows of maximum opportunity and impact. Although the epidemiological transition and associated risk factor transition conceptualize much of global health action in these terms, few studies or programs actually quantify the rise and fall of disease control opportunities over time. However, most health problems involve multiple or overlapping risk factors and sequelae, connected in complex and overlapping relationships, with trends often moving in opposite directions. Household air pollution offers just one important example of this phenomenon.

The CRA, by highlighting discrete risk factors that are associated with inevitably more complex temporal processes of change, may indirectly lead to the overvaluation of certain risk factors. Rather than rejecting this valuable approach, we have developed a method and interface for making such estimates more policy-relevant in light of ongoing processes of development [34,35]. Future iterations of the model can incorporate a broader range of diseases and risk factors, creating a dynamic tool for comparative risk analysis and priority setting. Such a tool could also account for cross-national variation in intervention effectiveness and second-order effects of health outcomes on social, economic and environmental outcomes. The IFs system of models is freely available to all at http://pardee.du.edu/access-ifs and the stove scenarios are included in Version 7.01 and beyond. We wish also to acknowledge our indebtedness to the GBD and CRAs for their pioneering work in identifying global health priorities and making this study possible.

## Supporting Information

S1 Appendix Methodological Appendix. (PDF)

S2 Appendix Bibliographic table for exposure estimation. (XLSX)

## References

[1] Lim SS, Vos T, Flaxman AD, Danaei G, Shibuya K, Adair-Rohani H, et al A comparative risk assessment of burden of disease and injury attributable to 67 risk factors and risk factor clusters in 21 regions, 1990–2010: a systematic analysis for the Global Burden of Disease Study 2010. Lancet. 2012;380: 2224–60. 10.1016/S0140-6736(12)61766-8 23245609 PMC4156511

[2] Smith KR, Bruce N, Balakrishnan K, Adair-Rohani H, Balmes J, Chafe Z, et al Millions dead: How do we know and what does it mean? Methods used in the comparative risk assessment of household air pollution. Annu Rev Public Health. 2014;35: 185–205. 10.1146/annurev-publhealth-032013-182356 24641558

[3] Murray CJ, Ezzati M, Lopez AD, Rodgers A, Vander Hoorn S. Comparative quantification of health risks conceptual framework and methodological issues. Popul Health Metr. 2003;1: 1–1. 12780936 10.1186/1478-7954-1-1 PMC156894

[4] Chafe ZA, Brauer M, Klimont Z, Van Dingenen R, Mehta S, Rao S, et al Household cooking with solid fuels contributes to ambient PM2.5 air pollution and the burden of disease. Environ Health Perspect. 2014;122: 1314–20. 10.1289/ehp.1206340 25192243 PMC4256045

[5] Brook RD, Rajagopalan S. The indoor-outdoor air-pollution continuum and the burden of cardiovascular disease: an opportunity for improving global health. Glob Heart. 2012;7: 207–213. 23181202 10.1016/j.gheart.2012.06.009 PMC3501678

[6] Brook RD, Rajagopalan S, Pope CA, Brook JR, Bhatnagar A, Diez-Roux AV, et al Particulate matter air pollution and cardiovascular disease: An update to the scientific statement from the American Heart Association. Circulation. 2010;121: 2331–78. 10.1161/CIR.0b013e3181dbece1 20458016

[7] Dherani M, Pope D, Mascarenhas M, Smith KR, Bruce N. Indoor air pollution from unprocessed solid fuel use and pneumonia risk in children aged under five years: a systematic review and meta-analysis. Bull World Health Organ. 2008;86: 390–402. 10.2471/BLT.07.044529 18545742 PMC2647443

[8] Kim K-H, Jahan SA, Kabir E. A review of diseases associated with household air pollution due to the use of biomass fuels. J Hazard Mater. 2011;192: 425–31. 10.1016/j.jhazmat.2011.05.087 21705140

[9] Lee M-S, Hang J, Zhang F, Dai H, Su L, Christiani DC. In-home solid fuel use and cardiovascular disease: a cross-sectional analysis of the Shanghai Putuo study. Environ Health. 2012;11: 1–8. 10.1186/1476-069X-11-18 22455369 PMC3349503

[10] Mishra VK, Retherford RD, Smith KR. Biomass cooking fuels and prevalence of tuberculosis in India. Int J Infect Dis. 1999;3: 119–29. 10460922 10.1016/s1201-9712(99)90032-2

[11] Pope CA, Burnett RT, Krewski D, Jerrett M, Shi Y, Calle EE, et al Cardiovascular mortality and exposure to airborne fine particulate matter and cigarette smoke: shape of the exposure-response relationship. Circulation. 2009;120: 941–8. 10.1161/CIRCULATIONAHA.109.857888 19720932

[12] Pope CA, Brook RD, Burnett RT, Dockery DW. How is cardiovascular disease mortality risk affected by duration and intensity of fine particulate matter exposure? An integration of the epidemiologic evidence. Air Qual Atmosphere Health. 2010;4: 5–14. 10.1007/s11869-010-0082-7

[13] Pope CA, Burnett RT, Thun MJ. Lung cancer, cardiopulmonary mortality, and long-term exposure to fine particulate air pollution. J Am Med Assoc. 2002;287: 1132–1142. 10.1001/jama.287.9.1132 PMC4037163 11879110

[14] Smith KR, McCracken JP, Weber MW, Hubbard A, Jenny A, Thompson LM, et al Effect of reduction in household air pollution on childhood pneumonia in Guatemala (RESPIRE): a randomised controlled trial. Lancet. 2011;378: 1717–26. 10.1016/S0140-6736(11)60921-5 22078686

[15] Lim SS, Vos T, Flaxman AD, Danaei G, Shibuya K, Adair-Rohani H, et al Supplementary appendix: a comparative risk assessment of burden of disease and injury attributable to 67 risk factors and risk factor clusters in 21 regions, 1990–2010: a systematic analysis for the Global Burden of Disease Study 2010. Lancet. 2013; 1–152. 10.1016/S0140-6736(12)61766-8 PMC4156511 23245609

[16] Global Burden of Disease. Global Burden of Disease Study 2010: results by risk factor 1990–2010 GBD 2010. Seattle, United States: Institute for Health Metrics and Evaluation; 2012.

[17] Bonjour S, Adair-Rohani H, Wolf J, Bruce NG, Mehta S, Prüss-Ustün A, et al Solid fuel use for household cooking: country and regional estimates for 1980–2010. Environ Health Perspect. 2013;121 10.1289/ehp.1205987 PMC3701999 23674502

[18] Smith KR, Ezzati M. How environmental health risks change with development: The epidemiologic and environmental risk transitions revisited. Annu Rev Environ Resour. 2005;30: 291–333. 10.1146/annurev.energy.30.050504.144424

[19] Wilkinson P, Smith KR, Davies M, Adair H, Armstrong BG, Barrett M, et al Public health benefits of strategies to reduce greenhouse-gas emissions: household energy. Lancet. 2009;374: 1917–29. 10.1016/S0140-6736(09)61713-X 19942273

[20] Hanna R, Duflo E, Greenstone M. Up in smoke: the influence ofhousehold behavior on the long-run impact of improved cooking stoves. Cambridge, MA: National Bureau of Economic Research; 2012 pp. 1–71. Report No.: 18033.

[21] Hughes BB. Forecasting long-term global change: introduction to International Futures (IFs). 2009;2009.12. Available: pardee.du.edu/sites/default/files/WP_2009_12_Introduction_IFs_0.pdf

[22] Hughes BB, Hillebrand EE. Exploring and shaping international futures. Boulder, Colorado: Paradigm; 2006.

[23] Hughes BB, Kuhn R, Peterson CM, Rothman DS, Solorzano JR, Mathers CD, et al Projections of global health outcomes form 2005 to 2060 using the International Futures integrated forecasting model. Bull World Health Organ. 2011;89: 478–486. 10.2471/BLT.10.083766 21734761 PMC3127264

[24] Hughes BB, Kuhn R, Peterson CM, Rothman DS, Solorzano JR. Improving global health: Forecasting the next 50 years. Boulder, Colorado: Paradigm Publishers; 2011.

[25] UNSTATS. Population using solid fuels, percentage. United Nations; 2013. Available: http://unstats.un.org/unsd/mdg/Metadata.aspx?IndicatorId=0&SeriesId=712

[26] WHO. Household fuel database. World Health Organization; 2013. Available: http://www.who.int/indoorair/health_impacts/he_database/en/

[27] Rothman DS, Irfan MT, Margolese-Malin E, Hughes BB, Moyer JD. Building global infrastructure: Forecasting the next 50 years. Boulder, Colorado: Paradigm Publishers; 2014.

[28] Balakrishnan K, Ghosh S, Ganguli B, Sambandam S, Bruce N, Barnes DF, et al Modeling national average household concentrations of PM2.5 from solid cookfuel use for the global burden of disease -2010 assessment: results from cross-sectional assessments in India. Environ Health. 2013;12: 77–77. 10.1186/1476-069X-12-77 24020494 PMC3851863

[29] Ezzati M, Kammen D. Indoor air pollution from biomass combustion and acute respiratory infections in Kenya: an exposure-response study. Lancet. 2001;358: 619–24. 11530148 10.1016/s0140-6736(01)05777-4

[30] Balakrishnan K, Parikh J, Sankar S, Padmavathi R, Srividya K. Daily average exposures to respirable particulate matter from combustion of biomass fuels in rural households of southern India. Environ Health Perspect. 2002;110: 1069–1075. 12417476 10.1289/ehp.021101069 PMC1241061

[31] Pillarisetti A, Mehta S, Smith KR. HAPIT, the Household Air Pollution Intervention Tool, to evaluate the health benefits and cost-effectiveness of clean cooking interventions Broken Pumps and Promises: Incentivizing Impact in Environmental Health. Springer International Press; 2016.

[32] Burnett RT, Pope CA, Ezzati M, Olives C, Lim SS, Mehta S, et al An integrated exposure-response function for estimating the global burden of disease attributable to ambient PM2.5 exposure. Environ Health Perspect. 2014;122: 43–43. 10.1289/ehp.1307049 24518036 PMC3984213

[33] Industrial Economics Incorporated. Particulate matter/mortality cessation lag. Uncertainty Analyses to Support the Second Section 812 Benefit-Cost Analysis of the Clean Air Act. Draft Report. Cambridge, MA; 2010. pp. 6,1–6,11. Available: http://www.epa.gov/cleanairactbenefits/may10/IEc_Uncertainty.pdf

[34] Omran AR. The epidemiological transition: a theory of epidemiology of population change. Milbank Mem Fund Q. 1971;49: 509–538. 5155251

[35] Smith KR. Environmental hazards during economic development: the risk transition and overlap In: Reichard E.G., Zapponi GA, editors. Assessing and Managing Health Risks from Drinking Water Contamination. Wallingford, UK: IAHS; 1995 pp. 3–13.

