# Supplementary material for: Beyond Attributable Burden: Estimating the Avoidable Burden of Disease Associated with Household Air Pollution
Source: PLoS One. 2016 Mar 16;11(3):e0149669. doi: 10.1371/journal.pone.0149669 (PMC4794200; doi:10.1371/journal.pone.0149669)
Supplement: S1 Appendix — (PDF) [file pone.0149669.s001.pdf]

Methodological Appendix for Beyond Attributable Burden: Estimating the avoidable burden of disease associated with household air pollution

The Comparative Risk Assessment in the GBD2010 Study

GBD 2010 included comprehensive estimates of deaths and DALYs attributable to 67 modifiable risk factors (59 individual risk factors grouped into 8 categories) for 291 causes of disease and injury.[1,2] The risk factor assessment was based on the calculation of population attributable risk (PAR) by cause, risk, country, age, and sex. First, for each cause of death and disability-adjusted life year (DALY) that is associated with a given risk factor, a population attributable fraction (PAF) was calculated, where a PAF was defined as the proportion of deaths or DALYs that would be eliminated if exposure levels were reduced to the theoretical minimum.

$$PAF = {(\sum RR(x)P(x) - \sum RR(x)P'(x))}\Big/{\sum RR(x)P(x)} = 1 - {\sum RR(x)P'(x)}\Big/{\sum RR(x)P(x)} \qquad (1)$$

where

> RR(x) is the relative risk associated with exposure level x; GBD (2010) assumed these to be the same across country, age, gender, and time period; P(x) is the population distribution in terms of exposure level, i.e. the shares of the population exposed to each level of exposure; and P'(x) is the theoretical minimum population distribution in terms of exposure level.

The disease-specific relative risks as a function of exposure levels were based on a systemic review and synthesis of published and unpublished literature. The distributions of exposure levels were estimated by country, gender, and age-group. Thus, PAFs were estimated for each risk-disease pair disaggregated by country, gender, and age-group.

The Population Attributable Mortality and DALYs for each cause, country, gender, and age-group were then calculated as the product of the PAF and the total mortality and DALYs for that cause

$$PAMortality = Mortality * PAF; PADALYs = DALYs * PAF$$

These were then summed across the various subcategories to produce aggregate results, e.g., total global mortality attributable to a given risk factor. Lim et al. (2012) present a number of these results for the years 1990 and 2010. We situate a temporal version of this approach into the the International Futures integrated forecasting system.

The International Futures System

The International Futures (IFs) simulation system is a structure-based, agent-class driven, dynamic modeling tool [3,4]. Figure 1 shows the major models in the system, all of which are linked in many ways that the figure cannot show. IFs draws upon standard modeling approaches from a wide range of disciplines, including population, economics, education, politics, agriculture, and the environment. For example, the demographic model incorporates a true cohort-component representation, tracking country-specific populations and events (including birth, death, and migration) over time by age and sex. IFs draws on an extensive database of indicators from all relevant disciplines.

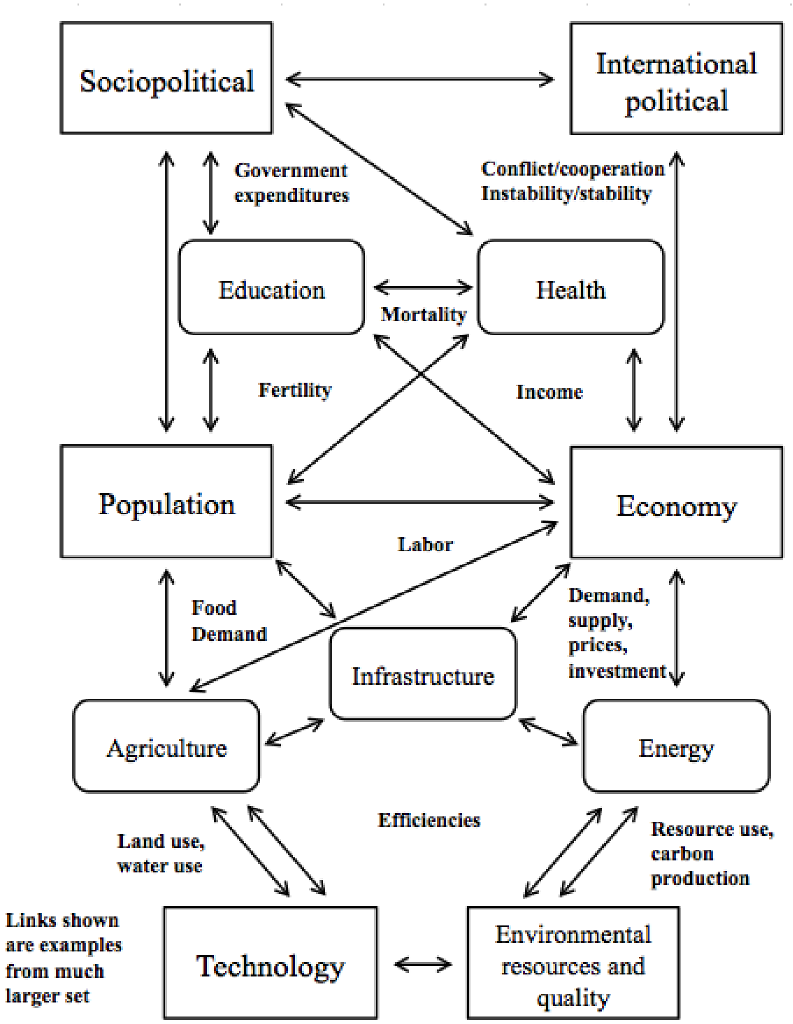

Figure A1 The major modules of International Futures (IFs)

The IFs health model uses distal drivers of socioeconomic change and proximate risk factors to forecast changing mortality and disease burdens [5,6] Data on 15 causes of death by country, age, and sex come from the World Health Organization's 2010 Global Health Estimates (GHE), and are broadly similar to the estimates constructed by the Institute for Health Metrics and Evaluation for the GBD 2010 study. We separated HAP-related causes of death out from their larger cause groups using data from a country's GBD subregion, based on the share of total deaths in the major cause accounted for by deaths in the smaller cause (specifically, ALRI was subdivided from respiratory infections, IHD and CVD from cardiovascular disease, COPD from respiratory illness, and lung cancers from malignant neoplasms). In all cases except lung cancers, the HAP-affected sub-cause accounted for a substantial share of the total cause group, thereby minimizing the potential error introduced by this assumption.

A forecast based on distal socioeconomic drivers builds on the World Health Organization's 2004 Global Burden of Disease forecasts, predicting age- sex- cause-specific mortality as a function of GDP per capita, Total Years of Adult Education (for adults 25 and older), a Smoking

Impact Factor, and time. Each of these distal drivers is forecast endogenously in the IFs system. In the base year (currently 2010), cause-specific estimates are normalized to fit GHE cause-specific values in the initial year. Age-specific death rates summed across all cause categories are then integrated into the larger cohort component population projection. Because initial fertility and migration estimates come from the UN Population Division (UNPD) World Population Prospects 2010 update, death rates are then normalized to UNPD all-cause age-specific death rates, with the cause-specific distribution preserved. As described in Hughes et al. (2011), we assessed our integrated mortality model through internal and external validation exercises against historical data and UNPD all-cause mortality forecasts.

After initialization, subsequent changes in cause-specific mortality rates are driven by the distal driver regressions and by a proximate risk factor adjustment.[1] The risk factor adjustment is critical to this analysis. It is based on a comparison of the actual PAF estimated for a population compared to the PAF that would have occurred if the risk factor were driven only by the distal drivers included in the mortality regression. The adjustment takes the following form:

$$\begin{aligned} Mortality_{Final} &= Mortality_{Distal} * \frac{1 - PAF_{Full}}{1 - PAF_{Distal}} \\ &= Mortality_{Distal} * \sum RR(x)P_{Full}(x) \,/ \sum RR(x)P_{Distal}(x) \end{aligned}$$

The full risk factor distribution is driven endogenously based on a more extensive set of drivers known to affect the risk factor, as described below in the case of liquid fuel cookstoves. The risk factor distribution can also be manipulated exogenously to create specific interventions such as a complete transition to liquid fuel cookstoves.

Finally, in order to calculate DALYs from deaths averted, we took advantage of the model's built-in cohort structure, which enables us to estimate not only how many people died, but also the ages at which they did so. Consistent with the 2010 GBD approach to calculating DALYs, we did not age-weight or discount our DALY estimates.

Stove forecast

The IFs model represents national trends in the use of modern stoves, including liquid, gaseous, and electric sources. Historical data on the percentage of households in each country primarily using solid fuels for heating and cooking were drawn from the 1990 to 2010 country-level dataset from UNSTATS and WHO used in GBD 2010 [7,8].[2] These data were used to initialize the model for the 2010 base year and to develop an equation used to forecast the expected shift towards increased use of modern stoves in the future in the absence of specific interventions.

---

[1] We explicitly model eight of the proximate risk factors of mortality identified in the CRA project: childhood underweight; body mass index; smoking; unsafe water, sanitation, hygiene; urban air pollution; indoor air pollution from household use of solid fuels; global climate change; and vehicle ownership and fatality rate.

[2] The model also represents current usage of efficient solid fuel stoves, building on estimates from the Global Alliance for Clean Cookstoves website http://www.cleancookstoves.org/resources/data-and-statistics/..Future papers will address models of partial emissions reduction through the distribution of such products.

This shift is assumed to follow a logistic function with the key driving factors being increases in average income, electricity access, and urbanization.[3]

Estimating HAP Exposure

To estimate the consequences of changes in HAP exposure, we conducted a review of the relationship between cookstove type and $PM_{2.5}$ exposure, drawing heavily on literature used by GBD 2010. We searched articles through Web of Science and Google Scholar (the latter to identify any grey literature). No formal date restrictions but emissions/concentration/exposure estimates papers were included back to 2000. Our searches for studies estimating solid fuel emissions and exposure or linking emissions to exposure used the following search terms

"cookstoves" OR "cook stoves" OR "Biomass stoves" OR "Household air pollution" OR "Indoor air pollution" AND "exposure" OR "emissions" OR "estimates" OR "concentration".

Additional searches on health effects included additional terms (particulate, PM2.5, effects, benefits, health COPD, ALRI, respiratory, cardiovascular, cancer).

Studies included in our estimation procedure measured concentration levels by stove type (not fuel type) in relation to $PM_{2.5}$ (or a measure that could be converted to $PM_{2.5}$) and provided evidence on variation exposure by different locations in the home. Table A2A at bottom provides a full list of papers reviewed including reasons for exclusion. We then estimated $PM_{2.5}$ exposure levels as a function of cookstove type, gender, and age, following the EPA guidelines for exposure assessments. We estimated $PM_{2.5}$ exposure levels as a function of cookstove type, gender, and age, following the EPA guidelines for exposure assessments, which are based on the following equation [9]:

$$E_C = \sum_{i=1}^{n} E_i * T_i$$

where:

$E_c = average\ daily\ exposure\ concentration\ for\ an\ individual$

$E_i = average\ daily\ exposure\ concentration\ in\ microenvironment\ i\ by\ cookstove\ type$

$T_i = fraction\ of\ 24\ hour\ day\ spent\ in\ microenvironment\ i$

We produced a range of estimates for exposure concentrations by fuel type building on past evidence from India and Kenya on 24-hour exposure concentrations by micro environment [10,11] and on age- and sex-specific exposure in relation to indoor time allocation. Based on these calculations, and with some qualitative assessment[4], we calculated separate exposure levels for women age 25+, men age 25+, and children under age 5 (these are the only population subgroupings for which there are estimates of health risk effects from indoor air pollution). We tested a wide variety of alternate base case exposure measures, which had minimal effect on our

[3] The specific form of the equation is $100/(e^{-z} + 1)$, where $z = 3{\cdot}40 - 0{\cdot}128 *$ average income $- 0{\cdot}037 *$ the percentage of population with access to electricity $- 0{\cdot}011 *$ the urban population percentage. The same equation was used to provide estimates for the year 2010 for countries for which data were not available.

[4] With their cooperation, our estimates were compared with the work of Dr. Kirk Smith and Ajay Pillarisetti at the University of California, Berkeley, who have developed a similar model. As their model has not yet been published, we will provide only general comparisons of their estimates with ours.

results. There was greater heterogeneity in the estimation of sex differences in exposure, and thus in our PAR results, and so we focus on all-sex findings.

Estimating Exposure-Response Relationships

We next converted exposure levels into disease-specific mortality risks based on the IER curves estimated for the GBD 2010 for ALRI, IHD, CVD, COPD, and lung cancers [12].[5] Table A1 presents the assumed exposure levels and disease-specific relative risks (RRs) by age group and sex for households using solid-fuel and modern stoves. Note that the RRs imply that solid fuel use elevates the risk for ALRI only for children under 5 and for other disease only for adults over 25.

Because it is unreasonable to assume that the health benefits of reductions in exposure to $PM_{2.5}$ are realized immediately after removing the source of exposure, we incorporated a lagged structure to assess health benefits. Our lag structure is in line with exposure assessment recommendations of the Environmental Protection Agency, which suggest calculating that 80% of the total health benefit accrues in the first five years after exposure ends [13]. We apply this recommendation in our model with different lag periods that are intended to capture the effects of differential timing of mortality benefits. Specifically, we assume that the reduction in ALRI risks happen within a year after the reduction in exposures; for CVD we assume this takes two years, and for pulmonary diseases our lag is five years [13].[6]

Table A1: Estimated particulate exposure and relative mortality risks by age, sex, stove type

Stove Type	Age/Sex Group	Exposure (pm2.5 / ug3)	Relative Risks				
			ALRI	COPD	IHD	Stroke	Cancer
Uses solid fuel	0-4, both sexes	200	2.62	1	1	1	1
	25+ female	200	1	2.34	1.39	1.51	1.9
	25+ male	70	1	1.42	1.31	1.43	1.4
Uses modern fuel	0-4, both sexes	7	1	1	1	1	1
	Female	7	1	1	1.02	1.01	1.01
	Male	7	1	1	1.02	1.01	1.01

Note: IHD and stroke risks reported for 80+; younger cohort risks are higher.

Works Cited

1 Lim SS, Vos T, Flaxman AD, *et al.* A comparative risk assessment of burden of disease and injury attributable to 67 risk factors and risk factor clusters in 21 regions, 1990-2010: a

[5] The one exception is COPD. The GBD authors were unable to use the IERs to estimate RRs for COPD and so used a fixed RR estimate based on their own meta-analysis, following the work of Kurmi et al. [14]

[6] This choice of lag structure was also informed by conversation with Dr. Kirk Smith and Ajay Pillarisetti on 11 November 2013 and via email.

systematic analysis for the Global Burden of Disease Study 2010. *The Lancet* 2012; **380**: 2224–60.

2 Lim SS, Vos T, Flaxman AD, *et al.* Supplementary appendix: A comparative risk assessment of burden of disease and injury attributable to 67 risk factors and risk factor clusters in 21 regions, 1990-2010: a systematic analysis for the Global Burden of Disease Study 2010. *The Lancet* 2013; : 1–152.

3 Hughes BB. Forecasting Long-term Global Change: Introduction to International Futures (IFs). 2009; **2009.12**.pardee.du.edu/sites/default/files/WP_2009_12_Introduction_IFs_0.pdf.

4 Hughes BB, Hillebrand E. Exploring and shaping international futures. Boulder, Colorado, Paradigm, 2006.

5 Hughes BB, Kuhn R, Peterson C, *et al.* Projections of global health outcomes form 2005 to 2060 using the International Futures integrated forecasting model. *Bull World Health Organ* 2011; **89**: 478–86.

6 Hughes BB, Kuhn R, Peterson C, Rothman D, Solorzano J. Improving Global Health. Boulder, Colorado, Paradigm Publishers, 2011.

7 UNSTATS. Population using solid fuels, percentage. 2013.http://unstats.un.org/unsd/mdg/Metadata.aspx?IndicatorId=0&SeriesId=712 (accessed 10 Oct2013).

8 WHO. Household Fuel Database. 2013.http://www.who.int/indoorair/health_impacts/he_database/en/ (accessed 10 Nov2013).

9 Mitchell KL, Smith RL, Murphy D. Estimating Inhalation Exposure. In: Air Toxics Risk Assessment Reference Library. Research Triangle Park, NC, US Environmental Protection Agency, 2004: 1–23.

10 Balakrishnan K, Ghosh S, Ganguli B, *et al.* Modeling national average household concentrations of PM2.5 from solid cookfuel use for the global burden of disease -2010 assessment: results from cross-sectional assessments in India. *Environ Health* 2013; **12**: 77–77.

11 Ezzati M, Kammen DM. Indoor air pollution from biomass combustion and acute respiratory infections in Kenya: an exposure-response study. *The Lancet* 2001; **358**: 619–24.

12 Burnett RT, Pope CA, Ezzati M, *et al.* An Integrated Exposure-Response Function for Estimating the Global Burden of Disease Attributable to Ambient PM2.5 Exposure Richard. *Environ Health Perspect* 2014; **122**: 43–43.

13 Industrial Economics Incorporated. Particulate Matter/Mortality Cessation Lag. In: Uncertainty Analyses to Support the Second Section 812 Benefit-Cost Analysis of the Clean Air Act, Draft Report. Cambridge, MA, 2010: 6,1–6,11.

14 Kurmi OP, Semple S, Simkhada P, Smith WCS, Ayres JG. COPD and chronic bronchitis risk of indoor air pollution from solid fuel: a systematic review and meta-analysis. *Thorax* 2010; **65**: 221–8.

Author	Date	Title	measure	outcome	included in study estimates	time use budget	multiple locations in home?	stove type
						specific inclusion criterion		
Ahuja et al.	1987	“Thermal Performance and Emission Characteristics of Unvented Biomass-Burning Cookstoves…	emissions	CO &TSP	no	no	no	no
Albalak et al.	2001	“Indoor Respirable Particulate Matter Concentrations from an Open Fire, Improved Cookstove, and LPG/open Fire Combination…	concentration	PM3.5	no	no	no	yes
Balakrishnan et al.	2002	“Daily Average Exposures to Respirable Particulate Matter from Combustion of Biomass Fuels in Rural Households of Southern India	concentration	respirable particulate matter	no	yes	yes	yes
Balakrishnan et al.	2004	Exposure Assessment for Respirable Particulates Associated with Household Fuel Use in Rural Districts of Andhra Pradesh, India	concentration	respirable particulate matter	yes	yes	yes	yes
Balakrishnan et al.	2013	“State and National Household Concentrations of PM2.5 from Solid Cookfuel Use	concentration	PM2.5	yes	no	yes	yes
Balakrishnan et al.	2013	“Modeling National Average Household Concentrations of PM2.5 from Solid Cookfuel Use for the Global Burden of Disease 2010	concentration	PM2.5	yes	no	yes	yes
Biran et al.	2007	Smoke and Malaria: Are Interventions to Reduce Exposure to Indoor Air Pollution Likely to Increase Exposure to Mosquitoes?	NA	malaria	no	no	no	no
Bonjour et al.	2013	Solid Fuel Use for Household Cooking: Country and Regional Estimates for 1980-2010	NA	% population using	no	no	no	no
Clark et al.	2013	Health and Household Air Pollution from Solid Fuel Use	concentration & exposure	PM10, PM4, PM2.5	no	no	no	yes
Ezzati and Kamman	2001	Indoor Air Pollution from Biomass Combustion and Acute	NA	health outcomes	no	no	nr*	yes

		Respiratory Infections in Kenya						
Ezzati and Kammen	2001	Quantifying the Effects of Exposure to Indoor Air Pollution from Biomass Combustion on Acute Respiratory Infections in Developing Countries	NA	health outcomes	no	nr	nr	yes
Ezzati et al.	2000	The Contributions of Emissions and Spatial Microenvironments to Exposure to Indoor Air Pollution from Biomass Combustion in Kenya	concentration	PM10	yes	yes	yes	yes
Ezzati et al.	2002	Review The Health Impacts of Exposure to Indoor Air Pollution from Solid Fuels in Developing Countries	expoure concentration	PM10	no	nr	nr	yes
Jetter and Kariher	2009	Solid Fuel Household Cook Stoves: Characterization of Performance and Emissions	emissions	CO, PM2.5	no	no	no	yes
Johnson et al.	2011	Modeling Indoor Air Pollution from Cookstove Emissions in Developing Countries Using a Monte Carlo Single-Box Model	emissions	PM2.5	no	no	no	yes
Joon et al.	2011	Predicting Exposure Levels of Respirable Particulate Matter (PM2.5) and Carbon Monoxide for the Cook from Combustion of Cooking Fuels	concentration	PM2.5	no	nr	nr	yes
MacCarty et al.	2010	Fuel Use and Emissions Performance of Fifty Cooking Stoves in the Laboratory and Related Benchmarks of Performance	emissions	CO, PM2.5	no	no	no	yes
McCraken et al.	2007	Chimney Stove Intervention to Reduce Long-Term Wood Smoke Exposure Lowers Blood Pressure among Guatemalan Women	personal exposure	PM2.5	no	no	no	yes
Meng et al.	2010	Determinants of Indoor and Personal Exposure to PM2.5 of Indoor and Outdoor Origin during the RIOPA Study	personal exposure	PM2.5	no	nr	no	no
Pokhrel et al.	2005	Case-Control Study of Indoor Cooking Smoke Exposure and	NA	cataract	no	no	no	no

		Cataract in Nepal and India						
Roden et al.	2009	Laboratory and Field Investigations of Particulate and Carbon Monoxide Emissions from Traditional and Improved Cookstoves	emissions	CO, PM	no	no	no	yes
Siddiqui et al.	2009	Indoor Carbon Monoxide and PM2.5 Concentrations by Cooking Fuels in Pakistan	concentration	CO, PM2.5	no	no	no	yes
Smith et al.	2010	Personal Child and Mother Carbon Monoxide Exposures and Kitchen Levels	personal exposure	CO	no	no	no	yes
Sukhosale et al.	2013	Indoor Air Pollution from Biomass Combustion and Its Adverse Health Effects in Central India	usage	health outcomes	no	no	no	no
Wilkinson et al.	2009	Public Health Benefits of Strategies to Reduce Greenhouse-Gas Emissions	NA	DALYs, deaths	no	no	no	no
Yadama et al.	2012	Social, Economic, and Resource Predictors of Variability in Household Air Pollution from Cookstove Emissions	NA	odds of ownership	no	no	no	no
Zhang et al.	2000	Greenhouse Gases and Other Airborne Pollutants from Household Stoves in China: A Database for Emission Factors	emissions	CO2, CO, CH4, TSP,NO4,SO2,K	no	no	no	no

* - nr = not reported, though it was used in the model
